# The Perception of South African Adolescents Regarding Primary Health Care Services

**DOI:** 10.1100/tsw.2006.161

**Published:** 2006-06-30

**Authors:** Magdalena S. Richter, Vivian Mfolo

**Affiliations:** ^1^ Faculty of Nursing, University of Alberta, Edmonton, Canada; ^2^ Department of Nursing Science, University of Pretoria, South Africa

**Keywords:** adolescent health, accessibility, primary health care services, South Africa

## Abstract

Most of the South African public health facilities fail to provide adolescent-friendly health services. A quantitative, descriptive research study was conducted at Stinkwater, a rural area in Hammanskraal, South Africa. The objective of the study was to describe the adolescent's preferences regarding primary health care services. A survey was conducted among 119 adolescents. It was found that adolescents wished to be involved in the planning of the activities of the adolescent health service, and that friendliness and respect for adolescents were seen as desirable characteristics of an adolescent-friendly health care service. Adolescents preferred services to be available throughout the week and to be located at the school, youth center, community center, hospital, or clinic. Health education was indicated as a priority and the health care team should include different members of a multidisciplinary team. Adolescents preferred that their health services be separated from adult services and that a male nurse be employed in the adolescent service in order to create a less feminine image. It was also recommended that all adolescents be educated about the types of services available. Understanding health care service preferences of adolescents is needed in order to deliver optimal health care to this group.

## INTRODUCTION

Accessibility of health care services is a principal tenant of all components of primary health care in South Africa. Access to health care is a priority regarding the health needs of adolescents as they experience physical, psychological, and social changes that may affect their health needs. Extensive research has established that most of the South African public health facilities fail to provide adolescent-friendly health services. Adolescents have to utilize existing primary health care services that are already overburdened. The growing demand for specialized health care services for adolescents is not being met by existing primary health care. Despite the fact that strategies for specific services are being introduced, the morbidity and mortality rates of adolescents continue to increase[1]. A recent review[2] indicated that variations of health care services to adolescents might be related to a combination of provider and patient factors and/or interaction among factors related to the health care system. Factors such as type and location of the clinic setting, general mistrust of the health care system, and concerns regarding confidentiality of the visit deter adolescents from seeking care. Health care factors include fragmented services and the failure to provide culturally sensitive and diverse workers. Other barriers that influence adolescents' use of primary health care include systems designed for young children or adults that are not responsive to adolescents' needs, legal restrictions on access, transportation problems, and lack of knowledge[3]. The needs and preferences of adolescents for primary health care services must be addressed in order to remove these barriers and increase access.

Health care providers spend limited time with the adolescent client and, therefore, can hardly address the above-stated issues. The vision of the South African health care plan that is to ensure that basic health care is available to all citizens is being defeated. Adolescent health care services are not included in most of the service programs. It is evident that there is a need to provide primary health care that caters to the special needs of adolescents. The purpose of this study was to describe the adolescent's preferences regarding primary health care services in a rural area in South Africa.

## METHODS

A quantitative descriptive design was used to obtain the adolescents' preferences regarding primary health care services. The target population was adolescents, aged 14–19 years, who attended health care services at a health care center, situated in the Stinkwater area in Hammanskraal, South Africa. Convenience sampling was used. Each adolescent who visited the clinic, and was willing to participate, was asked to complete a questionnaire. The questionnaire was developed after a thorough literature review that focused on the accessibility components of primary health services, the unique needs of adolescents, and the nature of services that would address their specific needs. The survey consisted of both closed and open-ended questions. Open-ended questions provided an opportunity for the adolescents to elaborate on closed-ended questions and allowed them to answer in any style and manner they preferred[4]. The adolescents were asked to indicate their preferences regarding the characteristics of a ***functionally accessible*** health service and they were asked to indicate the hours and days of the week that would be suitable to them to attend the health service. They were also asked about the ***geographical accessibility*** (e.g**.,** the clinic location) and their preferences regarding ***cultural accessibility*** (e.g., gender, age, and language of the health care provider), preferences regarding the availability of specific members of the health care team, types of service they would like to see included in the adolescent health service, and whether they had a need for health education. They were also asked to indicate if they were members of a health insurance/medical scheme ***(financial accessibility)***, and they were asked to indicate the amount they would pay for transport to the health service.

A pilot study was undertaken with the purpose of detecting possible flaws in the measurement procedures, to identify items not clearly formulated, and to allow the research team to take note of nonverbal behavior that might possibly signify discomfort with the content or wording of the questions[5]. During the pilot study, ten adolescents were approached and invited to complete the survey. The surveys were coded and analyzed to identify potential problems. These ten surveys did not form part of the main study. Neither the adolescents nor the research assistants experienced any problems in understanding the questions. The face validity of the survey was assessed and evaluated by an expert in the subject field. This expert independently evaluated the extent to which the survey addressed the most important issues mentioned in the literature. To ensure content validity, a peer group and colleagues of the researcher reviewed the survey. The reliability of the survey used in this study was confirmed by Cronbach's Alpha. A correlation coefficient higher than 0.70 was considered to be acceptable to demonstrate positive correlation. The correlation coefficients of the two main categories of the survey were determined and showed a positive correlation, which indicated reliability. Approval to conduct the study was obtained from the District Research Committee, District Manager, the Nursing Service Manager, and the Registered Nurse in charge at the center where the study was conducted. Participation was voluntary and informed consent was obtained from each participant. The adolescents' anonymity and confidentiality of the information were ensured.

The researcher trained two voluntary health care workers at the center to administer the surveys. Training focused on the sampling technique, the obtainment of consent, and assistance to the adolescents. The health care workers distributed 150 surveys over a period of 3 months; 119 surveys were completed before and on the cut-off date. The data were analyzed with the help of SAS Statistical software, version 8[6]. The open-ended questions were interpreted using thematic analysis.

## RESULTS

The results are tabulated according to demographic data as well as data about the functional, geographical, financial, and cultural accessibility of health care services. Of the adolescents, 83% were female, 42% were 14–16 years old, and 58% were 17–19 years old.

### Functional Accessibility

Fifty percent of the adolescents did not care whether the health care provider was a male or female. However, 39% of the adolescents indicated that they preferred a female health care provider, while 12% preferred to consult a male health care provider. Sixty-six percent preferred to go to young health care providers (age 20–29).

Fifty percent of the adolescents wished to be served in the language of their choice. Thirty percent of the adolescents did not have a language preference, while 20% preferred to be served in the language of the health care provider. Ninety-one percent preferred the presence of a doctor. It could be that they see the doctor as the main member of the health care team. A health promoter, social worker, and a nurse were also indicated as important members of the health team by a high percentage of adolescents. The current situation at health services in Hammanskraal is that the nurse is often the only available health team member. S/he has to attend to the problems of adolescent patients and can refer a patient to a doctor, who is only available on Mondays. Other members of the health care team are inaccessible to the adolescents because of physical distances from the health care services.

Fifty-three percent of the adolescents preferred to visit the clinic on a Saturday. Thirty-five percent indicated that they would like to attend the health service in the morning, while 27% would like to attend in the afternoon. Twenty-two percent would like the service to be open the whole day, while only 16% would prefer it to be a 24-h service. Forty percent preferred to be treated by a health care provider who specialized in all age groups, while 36% wished to see a health care provider who specialized in treating adolescents. Twenty-four percent of participants indicated that the specialty of the health care provider did not matter. Seventy-three percent of the participants preferred parental involvement, while 27% did not. The following reasons for noninvolvement were given: “When I am with my parents, I am not open hearted”, “They do not have time for me”, “They do not listen to me”, “Parents will never agree”, “I want privacy concerning my health issues”, “Parents will never be patient as I want to take my time at the health service”, “Father is always busy and Mother is too traditional”, “Because they will make me nervous”, “They are not honest with me”.

Ninety-nine percent of the adolescents preferred to be involved in the planning of the activities of the adolescent health service.

### Geographical Accessibility

Eighty-four percent of the respondents preferred to travel up to 5 km to the adolescent health service, while 11% stated that they were willing to travel 6–10 km. Only 5% were willing to travel more than 10

km to visit the adolescent health service. The context where this study was conducted could have influenced this response; it was a rural area and represented mainly a low socioeconomic population. The participants in this study most probably do not have the financial ability to pay for public transport. The response might be different in another population. Though the results are contextual, the mode of transport should be taken into consideration.

The adolescents were asked to indicate their preferences regarding the site of the adolescent health service. Ninety-six percent preferred the clinic as the site for an adolescent health service, while 87% would also be satisfied with an adolescent health service at a youth center.

## Financial Accessibility

Twenty-six percent of the respondents indicated that they were willing to pay for the services. Twenty-two percent indicated that they were aware, and had knowledge, of health service in the area.

## Cultural Accessibility

Forty-four percent of the adolescents indicated that the cultural and religious affiliation of the health care provider did not matter. A smaller percentage (27%) of participants preferred treatment by health care providers with the same cultural and religious background.

## A "Friendly" Health Service

The following open-ended question was included at the end of the survey: "Describe how you see an adolescent-friendly health care service." The characteristics as reflected by the participants are divided into three categories: (1) services, (2) staff characteristics, and (3) physical appearance of the staff.

1. The characteristics of an adolescent-friendly health care service were described as: "The center should not look like a clinic", "There is fun at the center with lots of activities", "Service is open 24 hours", "There are health education activities", "Service is concerned about the health of adolescents", "Service prepares adolescents for adulthood", "Service helps adolescents to face reality", "Availability of a hot line service", and "No long queuing."
2. Concerning the characteristics of the staff, the adolescents requested: "Nurses able to work with people", "Meet a health care provider that you know", "Health care provider that has knowledge and specializes in treating adolescents", "Doctor is open hearted and talks to everyone", "Health team member is friendly", "Personnel respect the adolescents", "Doctors have enough time for adolescents", "Good communication skills", "Staff have tolerance and patience", "Personnel are approachable", and "Supportive staff."
3. The adolescents described their requests concerning the physical appearance of the staff as: "Staffed with beautiful health care providers", "Smiling faces", "Dressed according to professional dressing codes", and "Nurses and doctors must look younger." Younger health care providers are seen as role models. Adolescents associate better with younger persons and feel that younger health care providers understand them better. This finding is supported by answers to an open question where several adolescents described the friendly adolescent health care service as one that is staffed by young personnel[1,7].

If taken into consideration, these views could promote user-friendly adolescent health services, thus enhancing accessibility.

## DISCUSSION

The purpose of the study was to describe the adolescents' preferences regarding primary health care services. Adolescents are a group with individual and neglected health care needs. Globally, there are a myriad of barriers that influence adolescents' use of health services. According to Juszczak et al.[3], these barriers include lack of confidentiality, systems designed for younger children, legal restrictions on access, transportation problems, lack of culturally appropriate services, and lack of knowledge, comfort, and interest by health professional in attending to their needs.

The results showed that female adolescents visited the clinic more often than their male counterparts. Male and female adolescents are equally vulnerable to health problems, although they develop at different rates and behave differently[1]. The WHO[1] stated that the gender inequities and differences that characterize social and economic life are reflected in the social organization of adolescents, and influence their health and development. In reproductive health, the difference in socialization and the power imbalance between the sexes heighten young women's vulnerability to negative health consequences, hence, a higher percentage of female adolescents who seek health care. The results could indicate that the programs were not gender sensitive. Studies by Marcell et al.[8] and Bekaert[9] have shown a decline in the use of health care services by older male adolescents (15–19 years) and the authors noted that there is a need to engage this group properly by establishing special male involvement programs.

The presence of different members of the multidisciplinary team at adolescent health care services is preferred. The WHO[1] has stated that a multidisciplinary health team is paramount for the effective provision of health services to adolescents. It is vital that these professionals are adequately trained to meet the needs of the adolescents effectively and sensitively. Adelman[10] agrees with this statement and says that adolescents are best served in adolescent clinics. The adolescents also indicated that they prefer a health promoter to be available. This is a valid demand, if seen against the background of the national health plan that has health promotion as one of its basic principles[11]. The national health plan emphasizes health education with the intent that adolescents should be empowered to make informed choices and decisions on matters relating to their health. The WHO[1] stated that the provision of an array of health services "under one roof" had been effective in meeting various needs of adolescents. Dennill et al.[11] stated that health education is viewed as the central tool in health promotion.

Although the adolescents in this study did not have a specific preference for the gender of the health care provider, a study that was conducted by Kapphahn et al.[12] in America reported that adolescent girls were more likely to prefer a same-gender health care provider than were boys. The adolescents in this study emphasized the need for health education. In another study[13], adolescents indicated a desire to have their provider discuss a variety of health issues. Health education services should be accessible as they increase adolescents' knowledge and understanding of a particular health issue and motivate them to adopt healthy behavior. Health promotion could prevent negative health behavior such as unwanted pregnancies, STDs, and substance abuse. Adolescents require information about specific potential risks to their health such as early-unprotected sex and abuse of alcohol and other drugs. All this information can be made available through accessible health education services[1].

The health care service should be available at hours that are convenient for adolescents. Richter[7] stated that the service should be available throughout the week and should include Saturdays. Stanhope and Lancaster[14] maintain that primary health care should include continuous coverage of 24 h/day and 7 days/week. Booyens[15] states that the provision of services on the days and hours that are convenient for the clients is another important aspect of accessibility. Adolescents need to be involved in the planning of the activities at the health care center. The WHO[1] supported these findings. It stated that involvement of adolescents in planning and implementing the activities of the adolescent health service would help to adapt the health service to the adolescents' special needs. Including adolescents' ideas and perspectives during program development can make the services more attractive and accessible to them.

The geographical area should be taken into consideration when a new service is planned. Dennill et al.[11] stated that health services should be within a reasonable walking distance (the WHO[1] suggested 5–10 km) and transportation should be available. Booyens[15] stated that the service provided must be convenient for the client in terms of transport and time constraints. In this study, the adolescents preferred an adolescent health care service to be located in a clinic or a hospital. It could be that adolescents realize that their needs can be met by a team of professionals who understands them[1]. Although youth and community centers are not health facilities, they could be ideal sites for an adolescent health service as these centers could provide health information, counseling, clinical services, and services that focus on the nonhealth needs of adolescents[1]. The school was also indicated as an acceptable site for an adolescent health service. Kibel and Wagstaff[16] agreed and stated that the school health services have the potential to reach a large section of the adolescents. A study conducted by Bekaert[9] indicates that an after-school drop-in center is a popular choice for adolescents. Adolescents should be informed where to find help if needed. Adolescents need to know and be aware of services being provided, their availability, and the way they are being rendered. Furthermore, the WHO[1] listed the barriers to the utilization of health services to adolescents such as services located a long distance away from where adolescents live/study/work or in places that are not easily accessible; services provided at times of the day when adolescents cannot get away from their study or work; and services provided at a place where people who know them, which would compromise discretion, could see them. Providing confidential care to adolescents is challenging. Akinbami et al.[17] suggested having a written policy on confidentiality when serving the adolescent population. Ongoing education about laws and regulations about confidentiality should be part of the policy.

Adolescents should not be excluded from services if they are unable to pay for the services. Dennill et al.[11] support these results and state that no person should be denied health care because of their inability to pay. The ability or inability of adolescents to pay for services and facilities is one of the factors that determined the accessibility or inaccessibility of health care[18].

Cultural sensitivity can have an influence on the utility of adolescent services. The basic respect for and sensitivity to the cultural and individual diversity of adolescents are features of successful programming approaches[1]. Programming, which demonstrates respect for cultural diversity, validates the personal worth of all adolescents and acknowledges the role that a sense of cultural identity plays in adolescent development. Van Rensburg et al.[18] agree that cultural preference is one of the factors that determined the accessibility of health care services.

The recommendations generated by study participants concerning clinical practice include:

- Adolescent health services should be separate from the services utilized by adults.
- A greater awareness of available youth health services must be created.
- Parents should have a positive attitude about the adolescent health services and should motivate the youth to utilize these services.
- Health planners must consider the decentralization of services to make them readily accessible to adolescents.
- More male health care workers should be employed in these services in order to lessen their feminine image.
- Boys should be encouraged to use these facilities.

There needs to be a reassessment of the availability of primary health care services for adolescents, especially in terms of accessibility. There should be further investigation to look into the feasibility of providing adolescent health services over weekends, especially for those adolescents attending school and/or working, and regarding the provision of clinics exclusively for adolescents, wherever possible, so that they need not fear encountering their parents at these clinics.

Policy makers and health care providers must allow more input from the adolescents themselves regarding their health needs in order to make services attractive to them and to facilitate the development of health services that are affordable, accessible, confidential, and nonjudgmental. Consideration must be given to the geographical and functional accessibility of primary health care services; adolescent health services should be separated from the adult services, and services should be rendered to adolescents at suitable times and by health care providers who are trained to work with them. It should be accessible in terms of cost, location, hours of operation, and the conditions laid down for usage. Taking account of local preferences and sensitivities regarding the age and sex of health care providers can also make a difference. By directing training efforts in adolescent health to the younger health care provider, many of these preferences noted by adolescents can be addressed successfully.

There were limitations to this study. The data may have been subject to sampling variability, as convenience sampling does not account for those adolescents who do not visit and access health care services. Thus, the preferences of adolescents from the present study are those only from participants who visited the clinic. Since the study was contextual in nature, results may not truly reflect the views of all adolescents in South Africa. Recommendations for future studies include carrying out a similar study on a larger scale in order to attain a better representation of the country's adolescents and include urban adolescents and those with disabilities. Results may not be generalized until national multisite investigations are performed. Nevertheless, this study provided a unique insight into adolescent health accessibility preferences and results have been consistent with other published studies.

The delivery of optimal health care to adolescents needs to address the differences in understanding service preferences. Even when health services are available, adolescents are often unable or unwilling to use them. Various barriers hinder the access of adolescents to these services and the utilization of health services[19]. The value of new research findings that cast new light on adolescents' preferences concerning access to appropriate health services cannot be overemphasized. Adolescents' health poses increasing reason for concern. Despite emphasis on client involvement and partnership in planning, little research has been attempted to demonstrate the achievements of the involvement of adolescents in the planning of health issues relating to them. The findings of this study can assist in the reconstruction and development of health care services in this specific area.

## ACKNOWLEDGMENT

The authors acknowledge the inputs from the participating adolescents, the voluntary health care workers that acted as data collectors, and the staff working at the Stinkwater Clinic at Hammanskraal, South Africa.

## BIOSKETCHES

**Magdalena S. Richter, DCur**, is an Assistant Professor at the Faculty of Nursing, University of Alberta, Canada. During the course of this study, she was a lecturer at the Department of Nursing Science, University of Pretoria, South Africa. Her primary research interest focuses on the health promotion of vulnerable groups within the family and/or those at vulnerable stages in family development. E-mail: solina.richter@ualberta.ca

**Vivian Mfolo, MCur**, was a master's degree student at the Department of Nursing Science, University of Pretoria during the course of the study. She is a part-time lecturer at the same Faculty. E-mail: nursing@med.up.ac.za
